# Analysis of Features Selected by a Deep Learning Model for Differential Treatment Selection in Depression

**DOI:** 10.3389/frai.2019.00031

**Published:** 2020-01-21

**Authors:** Joseph Mehltretter, Colleen Rollins, David Benrimoh, Robert Fratila, Kelly Perlman, Sonia Israel, Marc Miresco, Marina Wakid, Gustavo Turecki

**Affiliations:** ^1^Department of Computer Science, University of Southern California, Los Angeles, CA, United States; ^2^Department of Psychiatry, University of Cambridge, Cambridge, United Kingdom; ^3^Department of Psychiatry, McGill University, Montreal, QC, Canada; ^4^Faculty of Medicine, McGill University, Montreal, QC, Canada; ^5^Douglas Mental Health University Institute, Montreal, QC, Canada; ^6^Aifred Health, Montreal, QC, Canada; ^7^Department of Psychiatry, Jewish General Hospital, Montreal, QC, Canada

**Keywords:** deep learning, features, depression, interpretability, treatment

## Abstract

**Background:** Deep learning has utility in predicting differential antidepressant treatment response among patients with major depressive disorder, yet there remains a paucity of research describing how to interpret deep learning models in a clinically or etiologically meaningful way. In this paper, we describe methods for analyzing deep learning models of clinical and demographic psychiatric data, using our recent work on a deep learning model of STAR*D and CO-MED remission prediction.

**Methods:** Our deep learning analysis with STAR*D and CO-MED yielded four models that predicted response to the four treatments used across the two datasets. Here, we use classical statistics and simple data representations to improve interpretability of the features output by our deep learning model and provide finer grained understanding of their clinical and etiological significance. Specifically, we use representations derived from our model to yield features predicting both treatment non-response and differential treatment response to four standard antidepressants, and use linear regression and *t*-tests to address questions about the contribution of trauma, education, and somatic symptoms to our models.

**Results:** Traditional statistics were able to probe the input features of our deep learning models, reproducing results from previous research, while providing novel insights into depression causes and treatments. We found that specific features were predictive of treatment response, and were able to break these down by treatment and non-response categories; that specific trauma indices were differentially predictive of baseline depression severity; that somatic symptoms were significantly different between males and females, and that education and low income proved important psycho-social stressors associated with depression.

**Conclusion:** Traditional statistics can augment interpretation of deep learning models. Such interpretation can lend us new hypotheses about depression and contribute to building causal models of etiology and prognosis. We discuss dataset-specific effects and ideal clinical samples for machine learning analysis aimed at improving tools to assist in optimizing treatment.

## Introduction

The heterogeneity of depression constitutes a major barrier to successful treatment (Perna et al., 2018). Clinicians and patients are faced with a plethora of treatment options, with over 20 commonly prescribed antidepressants, augmentation therapies, psychotherapies, neuromodulation, and lifestyle interventions, but a paucity of evidence-based information to inform treatment selection and personalization. The resultant trial and error approach to treatment selection prescription is ineffective: a third of patients fail to remit to a first-line antidepressant, with remission rates decreasing with subsequent treatments (Rush et al., 2006). Researchers have strived to identify predictors of treatment outcome across clinical profile, sociodemographic, physiological, neuroimaging, genomic, and other possible predictor types (Williams et al., 2011), yet few, if any, predictors have translated into common clinical practice. Machine learning (ML) is capable of tackling the challenges of interpreting large, multidimensional, interrelated datasets found in psychiatric research and may help us create clinically useful models for treatment selection.

Two objectives in the study of biological systems are inference and prediction. Inference creates a model of data-generation to test a hypothesis about how a particular system behaves, whereas prediction forecasts possible outcome or behavior without necessarily understanding underlying biological mechanisms (Bzdok et al., 2018). Classical statistical methods, such as regression and *t*-tests, focus on inference and have been a dominant method for analyzing psychiatric data and offering insight into causal associations. For instance, logistic regression models assessing the association of demographic and clinical characteristics on treatment outcome in the Sequenced Treatment Alternatives to Relieve Depression (STAR*D) trial, a large multicenter sequenced treatment trial for depression, have shown that race, low education, post-traumatic stress disorder (PTSD), and hypochondriasis are independently associated with worsened depression (Friedman et al., 2009), as well as depression severity, energy/fatigue, race, education, and PTSD occurrence (Perlis, 2013); in addition, having witnessed or experienced trauma has been used to estimate risk for treatment-resistance among major depressive disorder (MDD) outpatients (Perlis, 2013). These results are bolstered with receiver operating characteristic (ROC) analyses also showing income and education to be predictors of response in STAR*D (Jakubovski and Bloch, 2014). However, in recent years, classical statistics and null hypothesis significance testing frameworks have been increasingly scrutinized due to the emphasis on *p*-value testing and difficulties with reproducibility (Wagenmakers, 2007). In contrast, machine learning allows for individualized prediction through the implementation of learning algorithms, which make fewer assumptions about data-generation, to find patterns in large, heterogeneous datasets. Advances in machine learning have highlighted its utility in identifying patterns in complex data for psychiatric research (Iniesta et al., 2016; Passos et al., 2016) and specifically for outcomes of depression treatments (Lee et al., 2018). Recent studies have leveraged machine learning methods to predict antidepressant treatment response for individuals with depression, identifying 25 features most predictive of whether a patient will respond to citalopram (Chekroud et al., 2016), predicting persistence, chronicity, and severity of depression from self-report questionnaires (Kessler et al., 2016), predicting treatment response to electroconvulsive therapy (ECT) using baseline hippocampal subfield volumes (Cao et al., 2018), predicting treatment resistance before initiation of a second antidepressant (Nie et al., 2018), using deep learning to predict response to SSRIs (Lin et al., 2018), and using Random Forests to predict outcome in treatment-resistant depression (Kautzky et al., 2018). However, the non-linearity of relationships that ML techniques capture in models make it difficult to integrate ML with existing biological knowledge and clinical practice, where researchers, clinicians, and patients often seek to understand causal relationships. We suggest that deep learning and traditional statistics can be used in a complementary fashion to interpret clinically meaningful associations.

One goal of personalized psychiatry is to predict a given patient's pre-treatment likelihood of response to an array of treatments in order to aid in selecting the treatment with the highest likelihood of response before therapy is administered. In recent work (Mehltretter et al., 2019), we performed a deep learning analysis on the Combining medications to enhance depression outcomes (CO-MED) clinical trial and Level 1 of STAR*D. Of all machine learning techniques, deep learning is considered one of the most effective, but also the most difficult to interpret (Zhang et al., 2018). We produced an algorithm that predicts response to four antidepressant treatments and is theoretically capable of increasing population remission rates via differential treatment benefit prediction (Mehltretter et al., 2019). Our study yielded four models, described below. As we examined each model's features found to be most predictive of remission, we identified striking consistencies in the features across models, and between our work and that of Chekroud et al. (2016) and others, as well as some surprising inconsistencies. Improving interpretability of deep learning models is important for translational research and for increasing their clinical utility. In our previous paper, we produced “interpretability reports” that helped understand the key features for predictions for individual patients. In this paper, we use regression and classical statistics to help interpret our results in order to better understand what complex ML outputs can tell us about the mechanisms driving remission to depression, and the relationships between predictive features. Based on these observations, we ask clinically- and mechanistically-relevant questions concerning general vs. specific predictors of response to antidepressants, trauma-related features, dataset differences in education, somatic symptoms and gender, using simple data representations and manipulations and traditional statistics, such as regression and *t*-tests. We evaluate our findings in the context of existing hypotheses concerning the etiology and prognosis of major depression and use what we learn to offer new directions for depression research and the use of ML in psychiatric data science.

## Materials and Methods

Here we discuss the data and models produced as part of our previous analysis (Mehltretter et al., 2019). We provide detailed methods in the Supplementary Methods section.

### Datasets

Data from CO-MED [Combining Medications to Enhance Depression Outcomes (COMED); ClinicalTrials.gov, NCT00590863] and STAR*D Level 1 (STAR*D; ClinicalTrials.gov, NCT00021528) were used for these analyses. CO-MED enrolled 665 outpatients who were randomly assigned three possible treatments: escitalopram and placebo, bupropion and escitalopram, or mirtazapine and venlafaxine. STAR*D Level 1 enrolled 2,757 subjects, all of whom were treated with citalopram.

### Feature Selection

A feature selection and analysis pipeline was used that consisted of variance thresholding, recursive feature elimination with cross validation, and feature importance extraction using a randomized lasso algorithm. The parameters for each method were optimized by analyzing the accuracy of the neural network's predictions about remission. Full details can be found in Mehltretter et al. (2019).

### Neural Network

A dense neural network was built with Vulcan (https://github.com/Aifred-Health/Vulcan) to train and evaluate our remission prediction capabilities. Since our data were limited in dimensionality we configured our neural networks to prevent over fitting by using a more shallow network. Each node within the network used scaled exponential linear unit function for activation, and softmax was used on the final layer for predicting the probability of remission.

### Models

We produced four models from different combinations of features from STAR*D and CO-MED, and compared these to a previously published model, and they are as follows:

1. **Combined model:** The combined model was developed by merging the STAR*D dataset (2,757 subjects, 1 treatment group) with the CO-MED dataset (665 subjects, 3 treatment groups) and removing features that were not common to both datasets, resulting in 3,222 patients, 4 treatment groups, and 213 features. We used variance thresholding and recursive feature elimination with cross validation to determine the features most salient for differential treatment prediction. This procedure identified 17 features.
2. STAR*D Optimal model: This remission-prediction model was trained on the citalopram data from level 1 of STAR*D, including all possible features in STAR*D without eliminating those not found in CO-MED, and was then validated using internal cross-validation.
3. STAR*D Tested on CO-MED: This model predicted remission with citalopram using features common to STAR*D and CO-MED, and generalized to the three branches of CO-MED to ensure our model wasn't biased toward citalopram.
4. **CO-MED Alone:** This model predicts remission for within the CO-MED dataset alone across all drug categories, including all the features present in CO-MED before feature selection. Six hundred and sixty five subjects were included and 25 features were used after feature selection for predicting remission.
5. **Chekroud et al. (****2016****) model:** We include results from the model detailed in Chekroud et al. (2016) to allow for direct comparison to our models. Chekroud et al. (2016) trained a gradient-boosting model on the citalopram data from level 1 of the STAR*D dataset and tested it on the three treatment groups of the CO-MED dataset, producing 25 features.

Table 1 demonstrates the features selected by the deep learning algorithm. Model performance metrics are reported in Table 2.

**Table 1:** Optimal features selected by the deep learning algorithm for remission prediction.

		**Model (number of features)**				
**Category**		**Combined (17)**	STAR*D optimal (21)	STAR*D tested on CO-MED (14)	**CO-MED alone (26)**	**Chekroud et al. (2016) (25)**
Sociodemographic		Number of years in formal education	Number of years in formal education	Number of years in formal education		Years of education
		Monthly household income	Monthly household income	Monthly household income		
						Black or African American
						White
			Current marital status			
			Months lived at residence			
			Has private insurance			
Patient history			Patient has a history of psychotropic meds		Previously taken zoloft sertraline	Ever taken sertraline
					Previously taken Prozac fluoxetine	
			Child history of depression			Number of previous major depressive episodes
Symptom profile (depression)	Depression severity	Initial QIDS total severity	Initial QIDS total severity	Initial QIDS total severity		Initial QIDS total severity
			Initial HAM-D depression severity			Initial HAM-D depression severity
		HAM-D suicide	QIDS suicidal ideation	QIDS suicidal ideation	QIDS suicidal ideation	HAM-D suicide
					Past 2 weeks: Considered hurting self or wished they were dead	
		QIDS mood (sad)	QIDS mood (sad)	QIDS mood (sad)		QIDS mood (sad)
						Depressed mood most of the day, nearly every day
	Somatic	HAM-D somatic energy		HAM-D somatic energy		HAM-D somatic energy
						HAM-D somatic anxiety
		Have you ever been bothered by aches and pains in many different parts of your body?	Have you ever been bothered by aches and pains in many different parts of your body?	Have you ever been bothered by aches and pains in many different parts of your body?	Have you ever been bothered by aches and pains in many different parts of your body?	Have you ever been bothered by aches and pains in many different parts of your body?
		QIDS weight (increase) last 2 weeks		QIDS weight (increase) last 2 weeks	Dysthymic disorder/major depressive episode. Weight loss or weight gain or appetite change	
		Eat a lot when not hungry			Feel disgusted after overeating	
	Sleep	QIDS sleep onset insomnia			Sleep onset insomnia	QIDS sleep onset insomnia
					I have been having more trouble sleeping than usual	HAM-D delayed insomnia
		QIDS energy or fatigability				QIDS energy or fatigability
	Cognitive or behavioral					QIDS psychomotor agitation
					QIDS concentration/decision making	
					Dysthymic disorder/major depressive episode: Poor concentration or difficulty making decisions	
						HAM-D loss of insight
					Feelings of worthlessness or guilt	
Comorbidity: Trauma		Have you ever witnessed a traumatic event such as rape, assault, someone dying in an accident, or any other extremely upsetting event?	Have you ever witnessed a traumatic event such as rape, assault, someone dying in an accident, or any other extremely upsetting event?	Have you ever witnessed a traumatic event such as rape, assault, someone dying in an accident, or any other extremely upsetting event?		Have you ever witnessed a traumatic event such as rape, assault, someone dying in an accident, or any other extremely upsetting event?
					Avoid activities that remind you of trauma	Did you try to avoid activities, places, or people that reminded you of a traumatic event?
	Jumpy because of a trauma	Jumpy because of a trauma	Jumpy because of a trauma		
		Did reminders of a traumatic event make you shake, break out into a sweat, or have a racing heart?				Did reminders of a traumatic event make you shake, break out into a sweat, or have a racing heart?
			Axis I: Post-traumatic stress disorder			
				Feel distant because of trauma	
Comorbidity: Anxiety		Anxiety being in crowded places	Anxiety being in crowded places	Anxiety being in crowded places		
			Did any of the following make you feel fearful, anxious, or nervous because you were afraid you'd have an anxiety attack in the situation? Standing in long lines	Did any of the following make you feel fearful, anxious, or nervous because you were afraid you'd have an anxiety attack in the situation? Standing in long lines		Did any of the following make you feel fearful, anxious, or nervous because you were afraid you'd have an anxiety attack in the situation? Standing in long lines
						Did any of the following make you feel fearful, anxious, or nervous because you were afraid you'd have an anxiety attack in the situation? Driving or riding in a car
					Avoid situation because afraid of anxiety attack	Did you have attacks of anxiety that caused you to avoid certain situations or to change your behavior or normal routine?
					Anxiety attacks for no reason	
Function			Current employment status			Currently employed
	How many hours did you actually work	How many hours did you actually work	How many hours did you actually work		
Symptom profile: Other			Neurological			
			Lower gastrointestinal (GI)			
					I talk more than usual	
					I suddenly feel very confident	
					I can feel my heart racing	
					Worry about saying something stupid	
					Worry about embarrassing self	
					Worry something you forgot	
					Guilt feelings and delusions	
					Hallucinations	
					Sleep disturbance	
Miscellaneous		Drug assigned			Assigned to randomization arm	

This table demonstrates the features composing each studied model. Note: for trauma, the following features were found to be predictive in STAR*D and CO-MED: “jumpy because of a trauma,” “ever witnessed a traumatic event,” and “Did reminders of a traumatic event make you shake, break out into a sweat, or have a racing heart?”.

**Table 2:** Ten-fold cross validated model accuracy metrics.

**Model (Number of features)**	**AUC**	**NPV**	**PPV**	**Sensitivity**	**Specificity**
Combined STAR*D + CO-MED (17)	0.69	0.64	0.64	0.60	0.60
STAR*D Optimal (21)	0.71	0.68	0.68	0.69	0.69
STAR*D Model that was then tested on CO-MED (14)	0.70	0.64	0.64	0.60	0.60
CO-MED Alone (26)	0.80	0.64	0.64	0.60	0.60
Chekroud et al. (2016) (STAR*D only) (25)	0.70	0.65	0.64	0.63	0.66

AUC, area under the curve; NPV, negative predictive value; PPV, positive predictive value; STAR*D, sequenced treatment alternatives to relieve depression; CO-MED, combining medications to enhance depression outcomes.

### Interpretation of Model Features

We set out to understand the features in these models and how they might relate to mechanisms of response in depression treatment and determination of initial depression severity, as this is an important predictor of response to treatment. We outline key observations from Table 1 that motivated five specific questions:

1. **Predictors of remission vs. predictors of response to specific antidepressants**
  By combining data from the STAR*D and CO-MED clinical trials for a pooled dataset across 4 treatments, we present a model that is able to perform differential treatment prediction. A benefit of this contribution is that we can begin to disentangle features that are predictive of remission regardless of drug category from features that are predictive of remission to specific drugs. We observed that two features were predictive of remission across all 5 models (Table 1): “Have you ever been bothered by aches and pains in many different parts of your body?” and suicidal ideation score. Their commonality across all models suggests that these are *general* predictors of response to antidepressant treatment, which reproduces some results from extant literature, in which suicidal ideation and somatic symptoms are robust contributors to more severe course of illness, increased rates of relapse, higher risk of suicide, and greater burden of care (Papakostas et al., 2003; Kapfhammer, 2006; Bohman et al., 2012). Four features—Number of years of formal education (beginning at grade 1), having witnessed a traumatic event initial depression severity [as assessed by Quick Inventory for Depressive Symptomatology (QIDS)], and sad mood (QIDS)—were common to all models except for the COMED-alone model. This suggests two non-mutually exclusive possibilities: that these represent citalopram-specific predictors of response, or that there were differences between the STAR*D and COMED samples, despite their large size and fairly broad inclusion criteria aimed at generating representative MDD samples. Given the possibility of antidepressant-specific vs. general predictors of response, we asked:
  “Can we identify features predictive of response to each of the four antidepressants within our model (escitalopram, bupropion, venlafaxine-mirtazapine, citalopram) individually, as well as to the subgroup of patients with a low probability of responding to any of the drugs?”
2. **Trauma-related features**
  Specific indices of trauma emerged from the deep learning model as predictive of treatment response for both the STAR*D and COMED datasets. Since trauma is also a strong risk factor for depression onset and severity (Nelson et al., 2017), this led us to question:
  “Are specific aspects of trauma predictive of baseline depression?”
3. **Differences in education level between datasets**
  While level of education was a feature that was relevant for predicting remission in STAR*D alone and in the combined dataset, it was not predictive in the CO-MED dataset alone. Since the combined dataset is biased toward STAR*D due to its larger sample size, this could explain the presence of the education feature the STAR*D and CO-MED combined dataset. We therefore analyzed the difference between levels of education for the two separate datasets to answer the question:
  “Do the participants in STAR*D and CO-MED come from the same population, or are these populations different in key variables that are predictive of outcomes?”
4. **Somatic symptoms and gender**
  Each of the four deep learning models retained somatic symptoms of depression, such as feeling aches and pains, as being important predictors of remission (Table 1). Gender, however, was not selected as an optimal feature predictive of remission. This could indicate that our model was not concerned with gender because it was able to extract specific features that differed between genders and therefore did not need to use gender as a proxy. Given that somatic symptoms have previously been shown to differ by gender (Silverstein et al., 2013), we asked:

“Do somatic symptoms of depression differ by gender?”

### Statistics

The data were analyzed at a Bonferroni-corrected significance level of *p* < 0.005 with the statistical software RStudio version 1.0.136. Statistical tests used were student's *t*-tests and linear regression.

## Results

### Can We Identify Features Predictive of Response to Each of the Four Antidepressants Within Our Model (Escitalopram, Bupropion, Venlafaxine-Mirtazapin, Citalopram) Individually, and Features Suggestive of a Low Probability of Responding to Any of the Drugs?

Given the four possible medications within our model, we assessed which features were important for predicting remission [as defined by a score of 5 or less on the Quick Inventory of Depressive Symptomatology (QIDS)] for each individual drug, as well as which features were predictive of a low probability of remission with any drug. We first defined a low probability of remission to any of the drugs as being a patient whose remission probability for each drug was less than the baseline population remission rate. This resulted in five sub-groups: one group for each of the four treatments, and a fifth group with a low probability of remission to any treatment. We created a set of 750 subjects: 500 randomly selected from the STAR*D study and 250 subjects randomly selected from the CO-MED trial. We assigned subjects to a sub-group by running our test set of subjects through our trained model four times, each time with a new medication storing the probability of remission for that given subject with that medication. We were, in effect, generating potential outcomes under each of four different treatments to see whether a patient would be predicted to experience remission under any or none of drugs. We then assigned each subject in a group based on the medication that produced the highest probability of remission. If no drug had a remission probability of greater than the baseline remission rate (34%), the patient was assigned to the non-remission group. This produced the following group sizes (Table 3).

**Table 3:** Number of subjects in each subgroup.

**Group**	**Number of subjects**
Non-remission	373
Escitalopram	28
Escitalopram-bupropion	28
Venlafaxine-mirtazapine	53
Citalopram	268

We then used saliency maps to identify the importance of each individual feature with regards to producing the given probability of remission, and took the top five for each subject. Tables 4–8 show how often a feature was found to be in the top five features for each sub-group, indicating the frequency, at the individual patient level, that this feature figured as one of the most influential features in the probability calculation.

**Table 4:** Non-remission subgroup feature information.

**Feature**	**% occurrence in top five**
Initial QIDS total severity	13.19
Have you ever been bothered by aches and pains in many different parts of your body?	13
Number of years in formal education	11.84
HAM-D somatic energy	9.71
QIDS energy or fatigability	9.33
Eat a lot when not hungry	7.27
QIDS sleep onset insomnia	6.1
Monthly household income	5.68
QIDS mood (sad)	5.68
Have you ever witnessed a traumatic event such as rape, assault, someone dying in an accident, or any other extremely upsetting event?	3.97
Jumpy because of a trauma	2.63
Anxiety being in crowded places	1.82
How many hours did you actually work	0.97
QIDS weight (increase) last 2 weeks	0.86
HAM-D suicide	0.75

*HAM-D, Hamilton Depression Rating Scale; QIDS, Quick Inventory of Depressive Symptomatology*.

**Table 5:** Escitalopram subgroup feature information.

**Feature**	**% occurrence in top five**
QIDS sleep onset insomnia	14.28
HAM-D somatic energy	13.57
Monthly household income	13.57
QIDS mood (sad)	13.57
Number of years in formal education	5.7
Jumpy because of a trauma	5
HAM-D suicide	5
How many hours did you actually work	5
Eat a lot when not hungry	1.43
QIDS energy or fatigability	1.43
Have you ever been bothered by aches and pains in many different parts of your body?	0.71
QIDS weight (increase) last 2 weeks	0.71
Initial QIDS total severity	0.71

*HAM-D, Hamilton Depression Rating Scale; QIDS, Quick Inventory of Depressive Symptomatology*.

**Table 6:** Escitalopram bupropion subgroup feature information.

**Feature**	**% occurrence in top five**
HAM-D somatic energy	14.29
Monthly household income	14.29
QIDS sleep onset insomnia	14.29
QIDS mood (sad)	14.29
Number of years in formal education	5.7
Jumpy because of a trauma	4.29
HAM-D suicide	4.29
How many hours did you actually work	4.29
Eat a lot when not hungry	1.43
QIDS energy or fatigability	1.43
Have you ever been bothered by aches and pains in many different parts of your body?	1.43
Initial QIDS total severity	1.43

*HAM-D, Hamilton Depression Rating Scale; QIDS, Quick Inventory of Depressive Symptomatology*.

**Table 7:** Venlafaxine-mirtazapine subgroup feature information.

**Feature**	**% occurrence in top five**
HAM-D somatic energy	14.33
Monthly household income	10.94
QIDS mood (sad)	10.94
QIDS sleep onset insomnia	10.57
Number of years in formal education	8.68
Initial QIDS total severity	6.8
Have you ever been bothered by aches and pains in many different parts of your body?	6.41
Have you ever witnessed a traumatic event such as rape, assault, someone dying in an accident, or any other extremely upsetting event?	3.77
Jumpy because of a trauma	3.01
How many hours did you actually work	3.01
QIDS energy or fatigability	2.64
HAM-D suicide	2.64
Eat a lot when not hungry	2.26
Anxiety being in crowded places	0.75

*HAM-D, Hamilton Depression Rating Scale; QIDS, Quick Inventory of Depressive Symptomatology*.

**Table 8:** Citalopram subgroup feature information.

**Feature**	**% occurrence in top five**
HAM-D somatic energy	14.25
QIDS mood (sad)	10.15
Monthly household income	10.15
Number of years in formal education	9.6
Initial QIDS total severity	8.28
Have you ever been bothered by aches and pains in many different parts of your body?	8.21
Have you ever witnessed a traumatic event such as rape, assault, someone dying in an accident, or any other extremely upsetting event?	4.2
QIDS energy or fatigability	4.2
Eat a lot when not hungry	4.1
Jumpy because of a trauma	1.72
How many hours did you actually work	1.57
HAM-D suicide	1.5
Anxiety being in crowded places	0.22
QIDS weight (increase) last 2 weeks	0.15
Did reminders of a traumatic event make you shake, break out into a sweat, or have a racing heart?	0.07

*HAM-D, Hamilton Depression Rating Scale; QIDS, Quick Inventory of Depressive Symptomatology*.

### Are Specific Aspects of Trauma Predictive of Baseline Depression?

We performed multiple linear regression analyses inputting the three trauma features deemed important by our deep learning model as predictors to explore the relationship between trauma and baseline QIDS score. One model included “jumpy because of traumatic event,” “witnessed traumatic event,” “shaky because of trauma,”; a second model also included gender and years of education as covariates. The linear regression models showed that only “Did reminders of a traumatic event make you shake, break out into a sweat, or have a racing heart?” was significantly associated with baseline depression severity, an association that remained after controlling for gender and years of education. Gender and years of education were also significantly predictive of baseline QIDS score (Table 9).

**Table 9:** Results of linear regression analyses examining the contribution of trauma indices to baseline depression severity in STAR^*^D.

	**Model 1**	**Model 2**
	**Beta estimate (S.E.) [95% CI]**	**Beta estimate (S.E.) [95% CI]**
Have you ever witnessed a traumatic event such as rape, assault, someone dying in an accident, or any other extremely upsetting event	0.246 (0.148) [−0.04, 0.54]	0.309 (0.171) [−0.03, 0.65]
Jumpy because of trauma	0.415 (0.177) [0.07, 0.76]	0.288 (0.20) [−0.108, 0.69]
Did reminders of a traumatic event make you shake, break out into a sweat, or have a racing heart?	1.027 (0.177) [0.68, 1.37]^*^	0.813 (0.202) [0.42–1.21]^*^
Gender		−0.794 (0.178) [−1.144, −0.44]^*^
Years of education		−0.098 (0.024) [−0.15, −0.05]^*^
F-statistic	30.00	17.97
*N*	2,696	1,951
R2	0.032	0.042

* *p < 0.005, the cut-off determined via a Bonferroni correction*.

### Do the Participants in STAR*D and CO-MED Come From the Same Population, or Are These Populations Different in Key Variables That Are Predictive of Outcomes?

**Figure 1:**
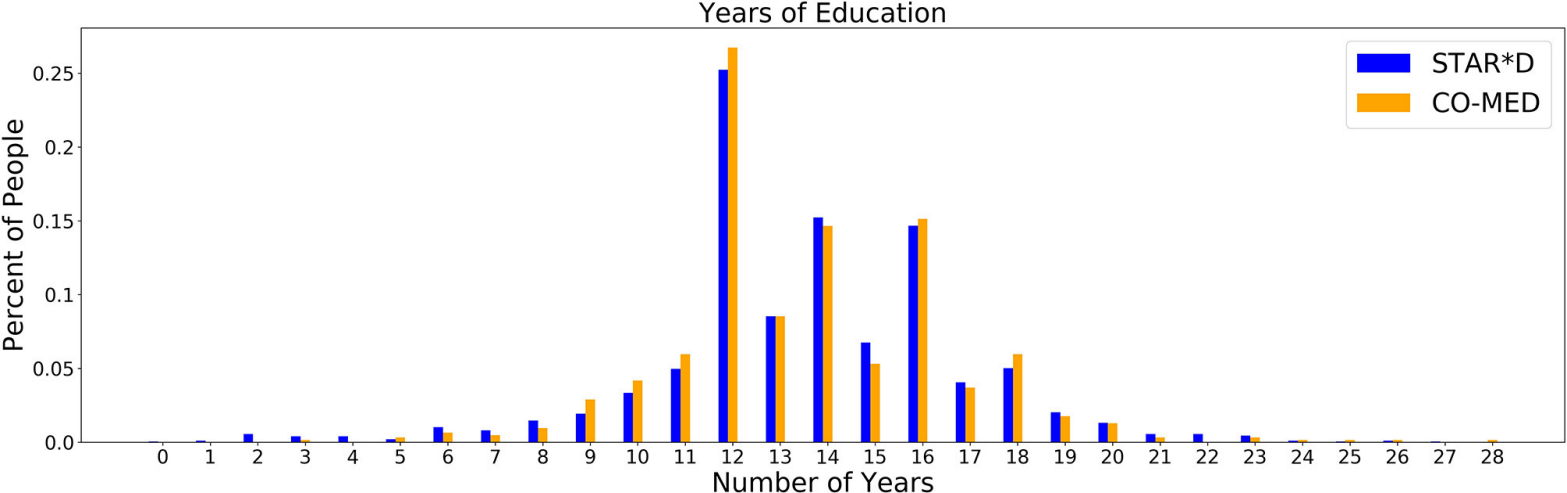
Number of years of education for the STAR*D and CO-MED datasets.

As education is more predictive of outcome in the STAR*D as compared to the CO-MED data, we performed independent *t*-tests to identify whether the distribution of education itself varied between participant samples, since education is unlikely to be a drug-specific predictor. As observed in Figure 1, a *t*-test showed there was no appreciable difference between the years of education in the CO-MED (orange bars) and STAR*D (blue bars) participants (mean difference = 0.06, *p* = 0.678).

### Do Somatic Symptoms of Depression Differ by Gender?

We used *t*-tests to see if somatic symptoms of depression differed between the genders. Table 10 details the difference in somatic symptoms between males and females, finding significant differences for the following features: somatic energy as measured by the Hamilton Depression Rating Scale (HAM-D), being bothered by aches/pains, and energy/fatigability, as measured by the Quick Inventory of Depressive Symptomatology (QIDS).

**Table 10:** Significant differences in somatic symptoms of depression between males and females.

	**Somatic energy**	**Bothered by aches/pains**	**Weight (increase) last 2 weeks**	**Energy/fatigability**
Male to Female Difference	−0.14^*^	−0.07^*^	−0.13	−0.21^*^
*p*-value	0.000	0.0001	0.027	0.000

* *p < 0.005, the cut-off determined via a Bonferroni correction*.

## Discussion

In this manuscript we analyzed the features retained by four deep learning models of depression treatment response. We show that traditional statistics can augment the interpretation of machine learning models, while informing the nature of the underlying datasets. In addition, we offer suggestions for optimizing future data collection to improve machine-learning analyses.

### Applying Insights From Machine Learning Features Toward Building Causal Mechanisms for Depression Pathology and Prognosis

#### Can We Identify Features Predictive of Response to Each of the Four Antidepressants Within Our Model (Escitalopram, Bupropion, Venlafaxine-Mirtazapine, Citalopram) Individually, as Well as to the Subgroup of Patients With a Low Probability of Responding to Any of the Drugs?

Across all four antidepressant subgroups, somatic energy was one of the most frequently observed features found to be in the top five features for each subject of that subgroup, consistent with previous machine learning approaches to predict response to antidepressant treatment (Chekroud et al., 2016). This may suggest that escitalopram, bupropion-escitalopram, venlafaxine-mirtazapine, and citalopram help alleviate energy symptoms (fatigue, heaviness in the body) more effectively than other symptoms. Indeed, a return of energy is often clinically observed early in treatment, and a similar effect can be observed for sad mood. Sleep-onset insomnia was also a strong predictor of response to escitalopram, bupropion-escitalopram, venlafaxine-mirtazapine, but not citalopram, suggesting that these antidepressants show some benefit in treating insomnia. However, insomnia has previously been associated with poorer treatment outcomes in some antidepressant trials (Sung et al., 2015), complicating our finding. Sleep interacts with stress to impact brain-derived neurotrophic factor levels (Giese et al., 2013), which are affected by certain antidepressants, and is also associated with other risk factors for depression, highlighting the complex interactions between depression symptomatology, risk factors like sleep, and the action of specific antidepressants. Household income was higher in the feature list of responders to each of the four antidepressant subgroups compared to the non-remission subgroup, suggesting that household income helps determine an individual's remission to any drug. This could reflect that lower income acts as a difficult-to-modify psychosocial stressor.

It should be noted that, between antidepressant categories, there were few striking differences in the symptoms more predictive of response to one treatment over another. This is consistent with the finding from the CO-MED study that there was equal efficacy of all three treatment arms. However, the model used in this analysis, detailed in Mehltretter et al. (2019), *did* find that differential treatment selection based on these features would be expected to improve population remission rates. That is, the study found that using a model trained on these features could usefully assign patients to different treatments, in a manner that suggests these treatments are not equally effective for all patients. This may be because of complex interactions between different *levels* of the different features. We may not be able to recover simple patient subtypes with the methods employed thus far. Instead, it may be the case that the subtypes that do exist include complex associations between multiple features. As a speculative example, the severity of sad mood and anxious symptoms, when combined with somatic symptoms, may have some value in determining which treatment may be most effective, over and above an analysis of the symptoms individually. We did not explore this here, but will address this question in future work. Another possibility for the lack of considerable differences in features reported in the different treatment subgroups (Tables 4–8) was the overall low number of features selected by the model. Though a low number of features was an efficient use of information when predicting remission, it was perhaps at the expense of losing some richness of explanation because it was mostly concerned with predicting remission with citalopram, the dominant drug class in the data.

We also identified features indicating a low probability of response to any of the drugs. Across all subjects with a low probability of response, initial depression severity most frequently emerged as the strongest predictor of non-response. This is consistent with extant research demonstrating increased depression severity is associated with non-response and treatment resistance (Berlim et al., 2008; De Carlo et al., 2016; Kautzky et al., 2017; Perlman et al., 2019). It suggests that the more severe the depression, the harder it will be to treat, regardless of the antidepressant. Number years education emerged as a drug-agnostic predictor of non-response. Considering its association with lack of remission (Perlman et al., 2019), low education appears an important psychosocial stressor that maintains depression, perhaps reflecting that, like low income, it is difficult to modify and therefore remains an ongoing factor that keeps people depressed for longer. Being bothered by aches and pains was also a general predictor of non-response, converging with current research on the alteration of somatic and interoceptive signaling in depression (Harshaw, 2015).

The identification of predictors of drug-specific response and general predictors of non-response to all types of treatment holds high clinical utility. Knowledge of which patients are unlikely to respond to any medication, and which will respond differentially to available first-line options will improve the treatment decision process. For instance, patients unlikely to respond to an antidepressant may consider adjunct psychotherapy, electroconvulsive therapy or intensive Day Hospital treatment earlier on in treatment, reducing prolonged symptoms of depression from ineffective treatments, potential side effects from medication, and wasted resources.

#### Are Specific Aspects of Trauma Predictive of Baseline Depression?

The regression analyses assessing the contribution of trauma measures to baseline depressive symptomatology found that trauma accounts for a significant proportion of the variance in baseline depression scores, with shakiness, sweating, or heart racing from trauma reminders, an indicator of a current physical reaction related to a past trauma, presenting as a stronger contributor to baseline depression than other trauma indices, such as ever having witnessed a trauma. This indicates that while experiencing trauma does confer some vulnerability, it is those who continue to manifest symptoms—those who may have some biological or other vulnerability to the prolonged effects of trauma—who have the most depressive symptoms, and therefore a lower chance to respond to treatment. Indeed, depression is highly comorbid with post-traumatic stress disorder (PTSD) (Flory and Yehuda, 2015), suggesting a trauma-related phenotype. However, neither STAR*D nor CO-MED excluded patients with PTSD, posing the limitation that our results might be driven by patients with concomitant PTSD. Further work is needed to explore our findings and potential links with the stress-diathesis model of depression (Monroe and Simons, 1991; Colodro-Conde et al., 2018). Gender was significantly associated with baseline depression severity, consistent with higher rates of depression in females, as was the number of years of education, suggesting that low education may be an important psychosocial stressor that contributes to and, as seen in the treatment resistance modeling of question (1) above, perpetuates depression.

#### Do Participants in STAR*D and CO-MED Come From the Same Population, or Are These Populations Different in Key Variables That Are Predictive of Outcomes?

Education was a significant predictor in the STAR*D trial, but not in COMED. We therefore assessed whether education levels differed between the datasets, but found no significant difference. Since education is unlikely to be a drug-specific predictor, we propose that even datasets that have broad inclusion criteria and that are traditionally considered “big data” by psychiatric standards, might not be large or diverse enough to capture all of the relationships of interest between sociodemographic variables and treatment outcome.

#### Do Somatic Symptoms of Depression Differ by Gender?

Our analysis of somatic symptoms showed that in comparison to males, females had lower somatic energy, were more bothered by aches and pains, and had increased fatigability. This reflects current research hypothesizing that gender differences in the prevalence of depression are due to increased somatic depression among females (Silverstein et al., 2013, 2017). This points toward not only the existence of specific subtypes of depression, but also toward testable hypotheses of mechanisms for such subtypes, such as increased susceptibility to inflammation in women (Derry et al., 2015). Our results equally converge with research on the hypothalamo-pituitary-adrenal (HPA)-axis response explaining the association between stress (trauma), pain (i.e., somatic symptoms), and fatigue (McEwen, 2007).

### Capturing Heterogeneity in Psychiatric Disorders: The Shift Toward “Big Diversity” in Patient Population Characteristics

Diagnostic entities in psychiatry are heterogeneous in nature, encompassing opposite ends of symptom dimensions. For major depressive disorder (MDD), diagnostic criteria can include weight gain or weight loss, increase or decrease in appetite, insomnia or hypersomnia, and psychomotor agitation or retardation (American Psychiatric Association, 2013). With 227 possible symptom combinations to meet a diagnosis of MDD (Zimmerman et al., 2015), two patients diagnosed with MDD may share no overlapping symptoms. This heterogeneity restricts the usefulness of psychiatric diagnoses for researching their etiology or prognosis, as different subtypes within a disorder might have different biological underpinnings and benefit from different types of treatment. Heterogeneity has not only hindered research, but may contribute to limited replication success in clinical trials (Dwyer et al., 2018). Traditional attempts to minimize or decompose heterogeneity include restricting inclusion criteria to focus on particular subgroups of patients (i.e., melancholic depression, treatment resistant depression, adolescent, or geriatric depression), either by imposing constraints on symptoms or limiting comorbidities, age, severity or chronicity of illness, in order to get obtain a “pure” or ideal sample of a certain subgroup to evaluate a priori hypotheses about that group. The problem with this approach is that it has not produced consistent subgroups (Marquand et al., 2016), the results may not generalize to independent samples, and such “ideal” patients are not representative of real-world heterogeneity. More optimal strategies for tackling heterogeneity may instead be data-driven approaches that capitalize on maximal heterogeneity in order to enhance generalizability of the model's predictions and mitigate bias. “Big data” requires not only large sample sizes, but “big diversity” in its samples, including multiple levels of data for each participant and variance in and across each type of data collected. Increasing data diversity will improve the generalizability and translatability of models and ensure that clinical decision aid tools might be more applicable to a broader range of individuals. Contrary to traditional approaches to experimental design in clinical populations, future research should explicitly capture variability, by including multiple study sites, ethnicities, socioeconomic levels, age, among others, to capture real-world variability and produce an ideal dataset for ML. This approach has been echoed by others and elaborated in the context of autism (Lombardo et al., 2019), but extends to all domains of mental health research. An important outcome of our deep learning model was that similar, but not identical feature sets were produced based on the sample used for training (STAR*D or COMED). For example, education, which is unlikely to be a treatment-specific predictor of response, was present in the STAR*D-dominated models, but not in the model that predicted remission in CO-MED alone, despite the average education level and the distribution of educational attainment not being significantly different between the two studies. While STAR*D was significantly larger than CO-MED, both of these datasets are considered to be large by psychiatric research standards. The fact that one of the most key features for predicting treatment response in one dataset was not predictive of treatment response in the other provides empirical support for advocating for larger and more diverse datasets. To optimize patient outcomes with precision psychiatry, the advent of “big data” necessitates a new focus on data with “big diversity.” The complexities of such data may be leveraged with ML approaches, and reinvestigated and understood with simpler, more interpretable models.

Our analyses exemplify how interpreting ML features can generate new hypotheses about disease pathology, contribute toward existing hypotheses, and help elucidate causal models which may have value in the development of new treatments or in treatment selection. Other efforts using a similar approach have proved equally fruitful: A recent study using a convolutional neural network to extract and quantify the relationship between features of the built environment and obesity prevalence showed that features of the built environment (i.e., greenery, different housing types, neighborhood density) were able to explain 64.8% of variation in obesity prevalence (Maharana and Nsoesie, 2018), demonstrating the utility of machine learning toward unpacking the association between the built environment and obesity prevalence. Through modeling complex interactions in “big data” samples, machine learning can uncover features associated with disease that can advance our understanding of psychiatric illnesses.

## Conclusion

The analytical power of machine learning is accompanied by limitations in its interpretability. In this paper we demonstrate the benefit of using traditional statistics to improve *post-hoc* interpretation of the features selected by deep learning models trained to predict remission in depression, and can provide a more meaningful clinical interpretation to understand interrelationships between important patient demographic and clinical characteristics and depression pathology. These approaches should be viewed as hypothesis generating and not confirmatory, as the “statistical significance” (*p*-values) associated with analyses performed on variables selected via ML or indeed any variable selection approach do not retain the standard interpretation. We emphasize the advantages of investing in “big diversity”—creating large and heterogeneous datasets, instead of the homogenous datasets favored by traditional large clinical studies—in order to produce datasets that are maximally useful for addressing important clinical questions.

## Data Availability Statement

Publicly available datasets were analyzed in this study. This data can be found using the NIMH data request and the following study identification numbers: NCT00590863; NCT00021528.

The Vulcan platform used for this work was open source and can be found here: (https://github.com/Aifred-Health/Vulcan). The data are available through the NIMH data request platform. Conflict of Interest Note: Note that we do not reproduce the final model in this work. This is because deep learning networks are difficult to represent in their final trained format, but also because the final model configuration is a trade secret of Aifred Health. However, since both the data and the platform used to train the model are open source, investigators should be able to produce versions of this model for further research purposes.

## Author Contributions

JM, DB, and RF contributed to the conception of the study and development of the deep learning models. JM and CR performed the statistical analyses. CR and JM wrote the first draft of the manuscript, with sections contributed by MW and DB. All authors contributed to manuscript revision, read, and approved the submitted version.

### Conflict of Interest

DB, KP, SI, RF, and MM are shareholders of Aifred Health, a medical technology company that uses deep learning to increase treatment efficacy in psychiatry. JM, MW, and CR have received consulting fees from Aifred Health. The remaining author declares that the research was conducted in the absence of any commercial or financial relationships that could be construed as a potential conflict of interest.
