# Supplementary material for: Analysis of Features Selected by a Deep Learning Model for Differential Treatment Selection in Depression
Source: Front Artif Intell. 2020 Jan 21;2:31. doi: 10.3389/frai.2019.00031 (PMC7861264; doi:10.3389/frai.2019.00031)
Supplement: Supplementary file 1 [file Data_Sheet_1.pdf]

Analysis of Features Selected by a Deep Learning Model for Differential Treatment Selection in Depression

Joseph Mehltretter[5]*, Colleen Rollins[6]*, David Benrimoh[2,3,4,7], Robert Fratila[7], Kelly[4,7] Perlman, Sonia Israel[4,7], Marc Miresco[1,7], Marina Wakid[4], Gustavo Turecki[2,4]**

[1]Department of Psychiatry, Jewish General Hospital, Montreal, Canada

[2]Department of Psychiatry, McGill University, Montreal, Canada

[3]Faculty of Medicine, McGill University, Montreal, Canada

[4]Douglas Mental Health University Institute, Montreal, Canada

[5]Department of Computer Science, University of Southern California, United States

[6]Department of Psychiatry, University of Cambridge, United Kingdom

[7]Aifred Health, Montreal, Canada

* J.M. and C.R. contributed equally to this paper.

**** Correspondence:**
David Benrimoh
david.benrimoh@mail.mcgill.ca

Keywords: deep learning, features, depression, interpretability, treatment

Supplementary Methods

In this section we describe the data pipeline which allowed us to create the four models described in this paper. Each model was produced in as similar manner, based on the methods described in Mehltretter et al., 2019.

We started by defining the dataset for each model. The Combined model was created from our combined dataset of 3,222 subjects, with only features common to CO-MED and STAR*D. The CO-MED alone and STAR*D optimal models were each created using the data from their respective studies, using the full feature lists from each study. The STAR*D tested on CO-MED model was created using data from STAR*D only, but with the feature set restricted to those common between STAR*D and CO-MED; the model trained on the STAR*D data was then tested on CO-MED data, which the reader should note acted as the hold-out set for this model.

Once the dataset for the model in question was defined, training and validation sets were created using an 80/20 split. In the combined model, an extra 200 patients were held out for the naïve analysis discussed in Mehltretter et al 2019 which is not discussed in this paper. We then sent the training data through our feature selection pipeline to produce our feature set for model training. Note that because of the 80/20 split, 20% of the data was not used for feature selection and this was the set later used for model testing. The feature selection pipeline consists of performing Recursive Feature Elimination with cross validation (RFECV) using three folds with a Random Forest Classifier. This method produced a subset of features considered the strongest in regards to predicting our target of remission. We then used our training set on the subset of features produced from RFECV to assess

the stability of those features using Randomized Lasso. This methodology takes random subsets of subjects and a random subset of features and runs a feature selection algorithm on that subset and selects the top features. It runs this process 200 times and upon completion it calculates the percentages a given features was selected as a top feature. We selected the features that were selected as top features 75% of the time or more. This resulted in the final feature sets for each model. Once a feature set was produced using our training data and our feature selection pipeline we performed our remission prediction analysis.

To first test the quality of the selected features, we performed a 10 fold cross-validation procedure on the whole dataset. This analysis did not produce a final trained model; rather it produced metrics for how well our features and model configuration predicted remission over a number of iterations of the model. These are the results presented in Table 2, to align with the Chekroud et al., 2016 results. This 10-fold cross validation process was entirely separate from the next step. We then trained final models and tested them on the held-out 20% of data which was held out during feature selection to assess performance of a model using the same features on a held out dataset which was not used during model training or feature selection (and in the case of the STAR*D tested on CO-MED model, these results were tested on the held-out CO-MED study). Model metrics on this hold-out set generally agreed with those produced during cross-validation. For the STAR*D optimal model, AUC on the 10-fold set and on the 20% hold out set were both essentially 0.7; for the CO-MED alone model AUCs for both analyses were essentially 0.8; for the Combined model the AUC's were essentially 0.69 in both analyses. For CO-MED tested on STAR*D, the model had an AUC of 0.7 on STAR*D, and between 0.63 and 0.64 AUC on each branch of CO-MED. These results indicate close agreement between the k-fold and held-out data tests. As noted, the data was imbalanced between remission and non-remission, reflecting clinical realities and the study results, and this extended to the randomly selected 20% hold-out sets. For the STAR*D optimal model, 23% of the hold out set were remitters; this was 18% for the CO-MED alone model and 25% for the Combined model. For the STAR*D tested on CO-MED model, remission rates varied by arm, between 37.7%-38.9%, as per the CO-MED study results. Data imbalance with respect to drug assigned was taken into account during the differential treatment benefit prediction step by ensuring that held-out data for the naive differential analysis reflected the underlying drug distribution, but this analysis is not discussed in this paper and is detailed in Mehltretter et. al, 2019. Future work will further consider the influence of balancing training, test, and validation sets on multiple variables, such as drug assigned and remission.

Supplementary References

Chekroud, A. M., Zotti, R. J., Shehzad, Z., Gueorguieva, R., Johnson, M. K.,Trivedi, M. H., et al. (2016). Cross-trial prediction of treatment outcome in depression: a machine learning approach. Lancet Psychiatry 3, 243–250. doi: 10.1016/S2215-0366(15)00471-X

Mehltretter, J., Fratila, F., Benrimoh, D., Kapelner, D., Perlman, K., Snook, E., et al. (2019). Differential treatment benefit prediction for treatment selection in depression: a deep learning analysis of STAR_D and CO-MED data. bioRxiv. Q19 doi: 10.1101/679779
